# The Works of Hippocrates and Galen

**Published:** 1845-10

**Authors:** 


### THE MEDICAL EXAMINER.

PHILADELPHIA, OCT., 1845.

☞ Several notices of new publications, accounts of cases, &c., designed for the present number, are unavoidably postponed, and will appear in our next.

---

We are gratified to find, that the learned and venerable Dr. J. R. Coxe, of this city, has prepared an epitome of the works of Hippocrates and Galen, which he proposes "to put to press if 500 subscribers of the thousands of medical men of the Union can be obtained. It is almost impossible to state precisely the extent of the work, derived as it is from seven or eight folios ; but it is believed that it can be so condensed as to be embraced in *three*, perhaps in *two*, octavo volumes, according to the type, of from 500 to 600 pages each, at a price not exceeding $3 a volume."

"I need not say"—Dr. Coxe states in his circular—"that it has been a work of considerable labour, yet assuredly one of infinite interest and gratification to myself; and it is chiefly from such considerations that I am induced to hope, that if printed, it will afford an equal gratification to my Medical contemporaries, and present to them, although epitomized,* an adequate idea of those venerable

* Although spoken of as epitomized, yet many of the Treatises of both the Authors, are fully tianslated.

writings, which have reached us after a lapse of more than two thousand years."

Every medical man, we presume, would desire to possess, in his library, the works of those venerable and venerated fathers of our art.

